# Icariin Attenuates Monocrotaline-Induced Pulmonary Arterial Hypertension via the Inhibition of TGF-*β*1/Smads Pathway in Rats

**DOI:** 10.1155/2020/9238428

**Published:** 2020-12-01

**Authors:** Yijia Xiang, Changhong Cai, Yonghui Wu, Lebing Yang, Shiyong Ye, Huan Zhao, Chunlai Zeng

**Affiliations:** Department of Cardiology, The Fifth Affiliated Hospital of Wenzhou Medical University, Lishui, Zhejiang 323000, China

## Abstract

**Background:**

Pulmonary artery remodeling is important in the development of pulmonary artery hypertension. The TGF-*β*1/Smads signaling pathway is activated in pulmonary arterial hypertension (PAH) in rats. Icariin (ICA) suppresses the TGF-*β*1/Smad2 pathway in myocardial fibrosis in rats. Therefore, we investigated the role of icariin in PAH by inhibiting the TGF-*β*1/Smads pathway.

**Methods:**

Rats were randomly divided into control, monocrotaline (MCT), MCT + ICA-low, and MCT + ICA-high groups. MCT (60 mg/kg) was subcutaneously injected to induce PAH, and icariin (50 or 100 mg/kg.d) was orally administered for 2 weeks. At the end of the fourth week, right ventricular systolic pressure (RVSP) was obtained and the right ventricular hypertrophy index (RI) was determined as the ratio of the right ventricular weight to the left ventricular plus septal weight (RV/LV + S). Western blots were used to determine the expression of TGF-*β*1, Smad2/3, P-Smad2/3, and matrix metalloproteinase-2 (MMP2) in lung tissues.

**Results:**

Compared to the control group, RVSP and RI were increased in the MCT group (*ρ* < 0.05). Additionally, TGF-*β*1, Smad2/3, P-Smad2/3, and MMP2 expressions were obviously increased (*ρ* < 0.01). Compared to the MCT group, RVSP and RI were decreased in the MCT + ICA group (*ρ* < 0.05). TGF-*β*1, Smad2/3, P-Smad2/3, and MMP2 expressions were also inhibited in the icariin treatment groups (*ρ* < 0.05)*. Conclusions*. Icariin may suppress MCT-induced PAH via the inhibition of the TGF*β*1-Smad2/3 pathway.

## 1. Introduction

Pulmonary arterial hypertension (PAH) is a serious vascular disease characterized by increased pulmonary artery pressure, progressive right heart hypertrophy, and heart failure [1]. Recently, PAH has become increasingly recognized as a chronic proliferative disease, particularly because of the extensive vascular remodeling of pulmonary artery vasculature, leading to intimal fibrosis, medial hypertrophy, luminal stenosis, and obliteration in small pulmonary arteries [2, 3]. Additionally, it is associated with poor prognosis, with a median survival time of 2 to 5 years from the point of diagnosis in most patients [4–6].

The transforming growth factor*-β*1 (TGF*-β*1) is an intercellular signaling molecule that binds to its receptors to transduce the message from the cell membrane to the nucleus [7, 8]. Additionally, TGF*β*1 regulates cellular proliferation, differentiation, and migration in various cell types [9–11]. It also induces the differentiation, migration, and apoptosis of pulmonary artery smooth muscle cells (PSMCs) in the media of pulmonary arteries [12–14]. In addition, the TGF-*β*1/Smad signaling pathway is activated during the PAH [15–18].

Icariin (ICA), isolated from *Epimedium pubescens*, is the main active flavonoid of *Herba Epimedii* [19, 20]. Additionally, it inhibits TGF*β*1/Smad2 signaling and alleviates myocardial fibrosis in rats [21]. Thus, this study was designed to elucidate the effects of icariin in PSMCs remodeling in PAH via the inhibition of the TGF-*β*1/Smads signal pathway.

## 2. Materials and Methods

### 2.1. Animals and Ethics Statement

In this study, male Sprague Dawley (SD) rats (aged 6–8 weeks, weighing 250–300 g) were obtained from the Laboratory Animal Center of Zhejiang province (certificate no. SCXL (Zhe) 2019-002). The animals were provided with free access to a standard diet and tap water at a temperature of 21 ± 1°C and a humidity of 55 ± 5%. The experimental procedures were approved by the Ethics Review of Animal Use Application of Fifth Affiliated Hospital of Wenzhou Medical University and were in accordance with the National Institute of Health Guidelines for the Care and Use of Laboratory Animals.

### 2.2. Monocrotaline (MCT) Treatment

MCT (Sigma, USA) was dissolved in 1 mol/L HCl, and the pH was adjusted to 7.20–7.40 with 1 mol/L NaOH. Rats in the MCT and MCT + ICA groups were subcutaneously injected with the MCT solution (diluted with 0.9% saline, 60 mg/kg) once. The control rats were injected with 0.9% saline (0.8 ml).

### 2.3. Treatment Protocol *In Vivo*

All the animals were randomly assigned to four groups. The control group rats (control, *n* = 10) orally administered 0.9% saline. Model group rats (MCT, *n* = 20) received MCT with 0.9% saline. Icariin group rats (*n* = 20/group) received icariin (Plant Bio-Engineering Co., Ltd., Xi'an, China) at a dose of 50 or 100 mg/kg per day. After 2 weeks of MCT injection, the rats in ICA groups were orally maintained daily for 2 weeks with different doses of ICA. Body weight was measured weekly to adjust the dose accordingly.

### 2.4. Hemodynamic and Cardiac Monitoring

Rats were anesthetized with isoflurane via the respiratory tract (induction with concentration of 5% for two minutes and a concentration of 2% for maintaining), followed by insertion of a catheter (PE50 tubule) into the right ventricular cavity through the right jugular vein. After measuring the right ventricular systolic pressure (RVSP, mmHg), the rats were sacrificed with 10% potassium chloride solution that was administered to the inferior vena cava under the anesthetized condition of isoflurane. The heart was dissected, and the right ventricular hypertrophy index (RI) was assessed by the ratio of the right ventricular weight to the left ventricular plus septal weight (RV/LV + S). Finally, the right lung was fixed in 10% formaldehyde for histopathology studies, and the remainder was stored at −80°C.

### 2.5. Histopathology

After incubation for 72 h, the upper lobe of the right lung tissues was dehydrated via a graded alcohol series, embedded in paraffin, and cut into 3–5 *μ*m thin sections. The tissue sections were stained with hematoxylin and eosin (HE) and Masson. Then, the small arteries (diameter 25–100 *µ*m) were visualized with a microscope. For each artery, the degree of wall thickness was calculated as follows: the ratio of the vascular wall thickness (WT%) = 100% × wall thickness/outer diameter. Three fields of six sections were randomly chosen for analysis in each group.

### 2.6. Immunohistochemistry

Lung tissue sections (4 *μ*m thick) were dewaxed and rehydrated and then washed with PBS (pH 7.2–7.4). After antigen retrieval and blocking with 5% bovine serum albumin (BSA), the sections were incubated with anti-*α*-smooth muscle actin (*α*-SMA) antibody overnight at 4°C, followed by the secondary antibody. Then, the sections were visualized with 3′3′-diaminobenzidine (DAB) and counterstained with hematoxylin. Afterwards, the stained sections were observed by the light microscope (Nikon, Japan).

### 2.7. Western Blot

Frozen lung tissues were used to extract the total protein. Bicinchoninic acid (Thermo, USA) reagent was used to measure the supernatant protein content. Extracts containing 80 *µ*g protein were electrophoresed and separated on 10% SDS-PAGE gels and transferred onto nitrocellulose membranes (Merck Millipore, Germany). The separated proteins were blocked with 5% skim milk at room temperature for one hour and incubated overnight at 4°C with primary antibodies, including MMP2 (diluted 1 : 1000; Abcam, England), TGF-*β*1 (diluted 1 : 1000; Abcam, England), Smad2 (diluted 1 : 1000; Abcam, England), Smad3 (diluted 1 : 5000; Abcam, England), P-Smad2 (diluted 1 : 300; Abcam, England), P-Smad3 (diluted 1 : 300; Abcam, England), and rabbit anti-GAPDH (diluted 1 : 1500; Cell Signaling Technology, USA). The membranes were then incubated with an HRP-conjugated secondary antibody (diluted 1 : 1000; Beyotime Institute of Technology, Shanghai, China) at room temperature for 1 h. The bands were detected using a Super Signal enhanced chemiluminescence (ECL) kit (Merck Millipore, Germany) in a western blot detection system (Bio-Rad, CA, USA) and quantified by density values, which were normalized to GAPDH.

### 2.8. Statistical Analysis

The data are presented as means ± SEM. Significant differences were determined by one-way ANOVA using GraphPad Prism (version 7.0) software followed by Tukey's multiple comparisons test. Values were considered to be significant when *ρ* < 0.05.

## 3. Results

### 3.1. Icariin Alleviated Pulmonary Artery Pressure and Right Heart Hypertrophy

**Figure 1:**
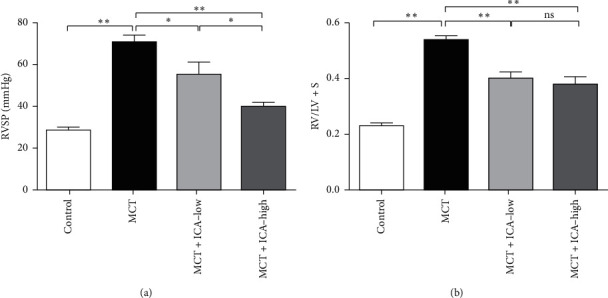
Icariin improves pulmonary artery pressure and right heart hypertrophy. RVSP and RV/LV + S were measured at the end of the 4^th^ week. Data are presented as mean ± SEM. ^*∗*^*ρ* < 0.05, ^*∗∗*^*ρ* < 0.01. *N* = 8/Control and *N* = 10/other groups.

At the end of the experiment, the RVSP was significantly increased in MCT-treated rats compared to the control group (70.51 ± 3.58 vs. 28.38 ± 1.26, *ρ* < 0.01), which indicated the successful induction of PAH by MCT, while RVSP was partially alleviated in the MCI + ICA-low group (55.60 ± 5.22, *ρ* < 0.05), and it was lower in the MCT + ICA-high group (39.75 ± 2.19, *ρ* < 0.01). Rats in the MCT group showed a significant right heart hypertrophy compared to the control group (0.54 ± 0.01 vs. 0.23 ± 0.01, *ρ* < 0.01), which was attenuated by icariin in the MCT + ICA-low (0.41 ± 0.02, *ρ* < 0.01) and MCT + ICA-high (0.38 ± 0.03, *ρ* < 0.01) groups. There were statistically significant differences in RVSP and RI among the four groups (Figure 1).

### 3.2. Icariin Suppressed Pulmonary Small Artery Remodeling

**Figure 2:**
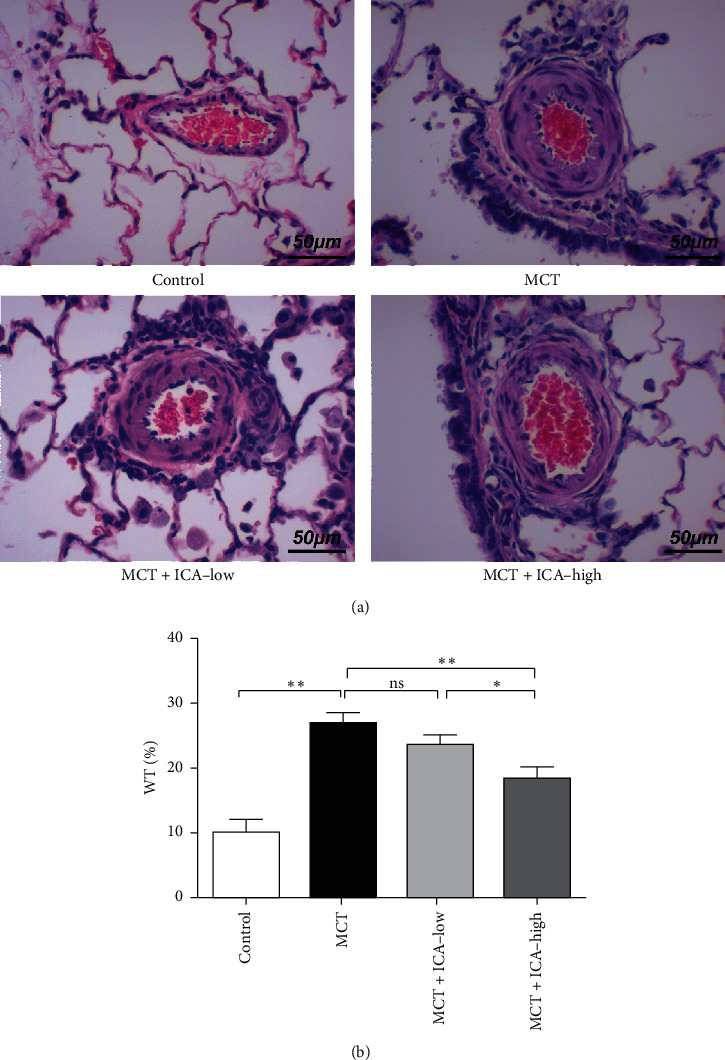
Icariin suppresses pulmonary small artery remodeling. Representative photomicrograph of pulmonary small artery remodeling indicated by HE staining (a) and WT% was used to measure the wall thickness in pulmonary small artery (b) (scan bar = 50 *µ*m, 400×). ^*∗*^*ρ* < 0.05, ^*∗∗*^*ρ* < 0.01, ns = no significant. *N* = 6/group.

**Figure 3:**
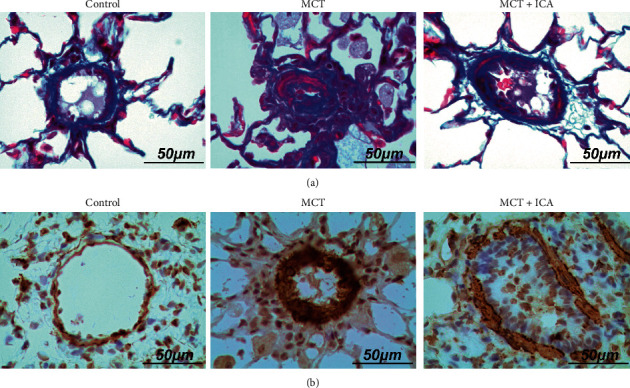
Icariin suppresses pulmonary small artery fibrosis. Representative photomicrograph of pulmonary small artery fibrosis indicated by Masson staining (a) and immunostaining with *α*-SMA (b) in the small pulmonary artery (scan bar = 50 *µ*m, 400×).

Under the microscope, the wall of small pulmonary artery was remarkably hypertrophic. Additionally, the WT (%) was obviously increased unlike those in the control group (*ρ* < 0.01). These pathological changes were improved with icariin treatment in the MCT + ICA-low group (*ρ* < 0.05) and MCT + ICA-high group (*ρ* < 0.01) (Figure 2). Additionally, treatment of icariin partly inhibited the fibrosis in PSMC with Masson stain (Figure 3).

### 3.3. Icariin Reduces Expression of TGF-*β*1 and Smad2/3

**Figure 4:**
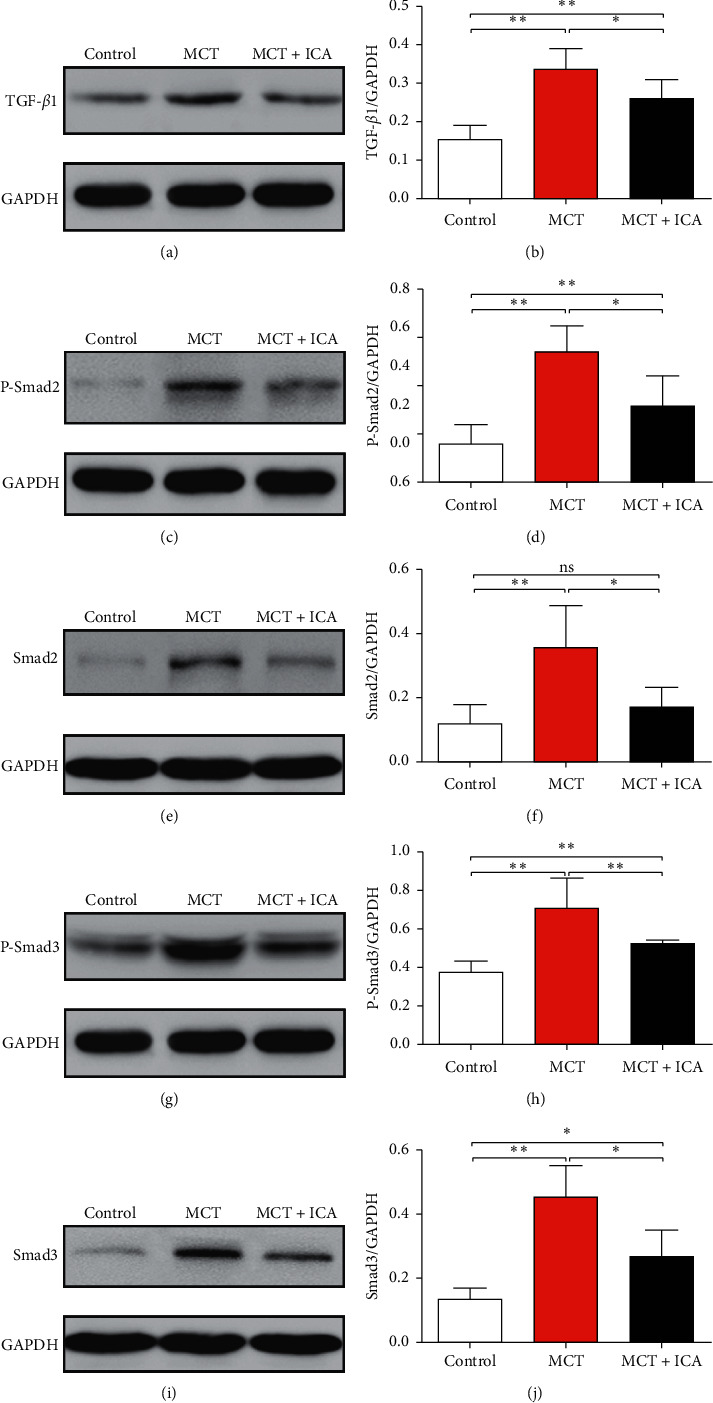
Icariin reduces TGF-*β*1, Smad2/3, and P-Smad2/3 protein levels in rat lung tissues. Western blot was performed to determine the expression levels of target proteins. ^∗∗^*ρ* < 0.01, ^∗^*ρ* < 0.05, ns = no significant, *N* = 6/group (P-Smad3, Smad3, *n* = 4/group).

The results showed that MCT injection caused a significant increase in the TGF-*β*1, Smad2, P-Smad2, Smad3, and P-Smad3 protein levels. The level of TGF-*β*1 was 0.33 ± 0.02, as compared to the control (0.15 ± 0.01, *ρ* < 0.01, *n* = 6/group). The Smad2 expression was increased from 0.12 ± 0.02 in the control to 0.35 ± 0.05 (*ρ* < 0.01, *n* = 6/group), and P-Smad2 increased from 0.15 ± 0.04 in the control to 0.54 ± 0.04 (*ρ* < 0.01, *n* = 6/group). The Smad3 level was 0.45 ± 0.05 in the MCT group and 0.13 ± 0.02 in the control group (*ρ* < 0.01, *n* = 4/group), and P-Smad3 was 0.71 ± 0.07 in the MCT group and 0.37 ± 0.03 in the control group (*ρ* < 0.01, *n* = 4/group). Icariin treatment partially suppressed the expression of TGF-*β*1, Smad2/3, and P-Smad2 proteins when compared to MCT injection alone (0.26 ± 0.02, 0.17 ± 0.02, 0.27 ± 0.04, and 0.30 ± 0.05, respectively, *ρ* < 0.05). Additionally, P-Smad3 was also decreased in the MCT + ICA group (0.53 ± 0.01, *ρ* < 0.01, Figure 4). However, these changes were not down to normal.

### 3.4. Icariin Inhibits Expression of MMP2

**Figure 5:**
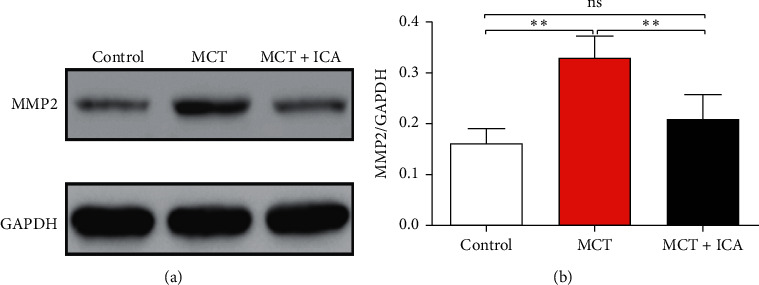
Icariin inhibits MMP2 protein level in rat lung tissues. Oral administration of icariin suppressed MMP2 expression. ^∗∗^*ρ* < 0.01, ns = no significant, *N* = 6/group.

Our results show increased levels of MMP2 in the MCT group from 0.32 ± 0.02 to 0.16 ± 0.01 in the control group (*ρ* < 0.01, *n* = 6/group) by western blotting. Oral administration of icariin inhibited the expression of MMP2 when compared to the MCT injection alone (0.21 ± 0.02 vs. 0.32 ± 0.02, *ρ* < 0.01, *n* = 6/group; Figure 5).

## 4. Discussion

PAH is a malignant vascular disease with poor prognosis. In our study, compared with the control group, RVSP and RI in the MCT group were significantly increased, indicating that the model of MCT-related PAH was successfully established. In this study, treatment with a different dose of icariin markedly suppressed pulmonary small artery remodeling, which ameliorated pulmonary hypertension and right heart hypertrophy. In addition, previous studies demonstrated that progressive thickening of the pulmonary artery wall contributed to the development of PAH [22–25]. Therefore, the thickness of the vascular wall was observed by the HE stain and WT%. Additionally, it was proved that WT% was strongly increased in the MCT group, and treatment with icariin improved WT% in a dose-dependent manner. Thus, these results show that the inhibiting effect of icariin on PAH may be related to dose. Thus, treatment with icariin with a dose of 100 mg/kg·d was used to analyse the target proteins and to observe the changes in the small pulmonary artery by Masson stains, as well as *α*-SMA by immunohistochemical assay. Studies have shown that smooth muscle cell (SMC) and endothelial cell (EC) proliferation are involved in the pathology of pulmonary vascular remodeling in pulmonary hypertension [26–28]. In this study, we observed the media change in the small pulmonary artery with *α*-SMA, in accordance with the HE stain. Additionally, treatment with icariin reduced TGF-*β*1, Smad2/3, P-Smad2/3, and MMP2 expression.

The members of TGF-*β* family cause numerous cellular responses through different receptors and intracellular signal pathways and are significant mediators in pulmonary fibrosis and vascular remodeling [8, 29–32]. TGF-*β*1, which is one of three isoforms, is the most abundantly expressed one [33]. TGF-*β*1 binds to type I and II receptors on the cell surface, and the intracellular signaling induced by TGF-*β* ligands is then mediated by the Smad family proteins. Smad2 and Smad3 are the first identified substrates of the TbRI kinase, which are activated through carboxy-terminal phosphorylation. The receptor-activated Smads (R-Smads) are released from the receptor complex to form a heterotrimer of two R-Smads and one Smad4, which then translocates to the nucleus to regulate target gene expression [29]. As in previous studies, in the model of the MCT-induced PAH, the relative levels of TGF-*β*1, P-Smad/Smad2, and P-Smad3/Smad3 were increased significantly in lung tissues [34, 35]. These results indicated that the TGF-*β*1-Smad2/3 signal pathway was involved in the process of PAH. Additionally, treatment with icariin partly reduced the levels of these target proteins in lung tissues. Thus, we may speculate that icariin may improve the PAH via the inhibition of TGF-*β*1-Smad2/3 signal pathway at the molecular level, but it will be confirmed *in vitro* and *in vivo* research studies in the future.

ECM represents a key component of pulmonary vascular remodeling and regulates the metabolism by the matrix metalloproteinases (MMPs). Imbalance in MMPs and ECM metabolism induces pulmonary hypertension. TGF-*β*1 increases ECM deposition during vascular remodeling, mainly collagen type I and fibronectin deposition [36, 37]. Moreover, TGF-*β*1, Smad2, and Smad3 can increase the synthesis of fibronectin, collagen, and proteoglycans and can reduce the decomposition of collagen protein, further resulting in an imbalance of the extracellular matrix and the deposition of collagen. Further, extracellular TGF-*β*1 may be activated by proteases, such as MMP2 [38]. Our results show that the lung tissues of MCT-induced rats display a higher expression of MMP2. This result demonstrates the complex interactions between the TGF-*β*1/Smad2/3 pathways and ECM metabolism in the pathology of vascular remodeling in MCT-induced PAH. In addition, treatment with icariin suppressed the expression of MMP2 in lung tissues. Additionally, it indicated that icariin may inhibit the complex reaction between TGF-*β*1/Smad2/3 pathway and MMP2.

## 5. Conclusion

In conclusion, our study showed that icariin ameliorated MCT-induced pulmonary hypertension. The possible mechanism is likely mediated via the suppression of TGF-*β*1/Smad2/3 signaling.

## Data Availability

The data used to support the findings of this study are available from the corresponding author upon request.
